# Nutritional Composition, Technological Quality, and Sensory Attributes of Chicken Breast Meat Affected by White Striping, Wooden Breast, and Spaghetti Meat: A Comprehensive Evaluation

**DOI:** 10.3390/foods13244007

**Published:** 2024-12-11

**Authors:** Marija Bošković Cabrol, Gerolamo Xiccato, Massimiliano Petracci, Pilar Hernández Pérez, Christine Mayr Marangon, Angela Trocino

**Affiliations:** 1 Department of Agronomy, Food, Natural Resources, Animal and Environment (DAFNAE), University of Padova, 35020 Legnaro, Padova, Italy; gerolamo.xiccato@unipd.it (G.X.); christine.marangon@unipd.it (C.M.M.); angela.trocino@unipd.it (A.T.); 2 Department of Food Hygiene and Technology, Faculty of Veterinary Medicine, University of Belgrade, Bulevar Oslobodjenja 18, 11000 Belgrade, Serbia; 3 Department of Agricultural and Food Sciences, Alma Mater Studiorum, University of Bologna, 47521 Cesena, Forlì-Cesena, Italy; m.petracci@unibo.it; 4 Institute for Animal Science and Technology, Universitat Politècnica de València, 46022 Valencia, Spain; phernan@dca.upv.es; 5 Department of Comparative Biomedicine and Food Science (BCA), University of Padova, 35020 Legnaro, Padova, Italy

**Keywords:** growth-related myopathies, texture, fat quality, amino acid content, minerals

## Abstract

This study assessed the impact of growth-related myopathies—white striping (WS), wooden breast (WB), and spaghetti meat (SM)—on the technological properties, lipid and protein oxidation, chemical composition, and profiles of fatty acids (FAs), amino acids, minerals, and sensory attributes of *pectoralis major* muscles in broiler chickens. Breasts with myopathies had similar pH and lightness but exhibited lower redness and yellowness in the case of WB defect compared to normal meat (*p* < 0.05). The WB samples also showed higher cooking losses than normal meat and increased shear force compared to the SM samples (*p* < 0.01). Moreover, WB meat showed lower protein content (*p* < 0.001) than the normal and SM samples but the highest glycine content (*p* < 0.05). The WB meat also exhibited significant alterations in FA composition, with higher levels (*p*< 0.05) of C18:2n6, C22:6n3, n3 PUFA, n6 PUFA, and total PUFA compared to the normal and SM samples. The presence of myopathies did not influence the meat mineral composition, oxidative pattern, or sensory properties. In conclusion, growth-related myopathies in broiler chickens impact the technological quality and chemical composition of their breast meat, with WB showing the most significant alterations in protein content and FA composition. These changes indicate potential challenges to processing and nutritional quality, though sensory attributes remain largely unaffected.

## 1. Introduction

The intensification of poultry production and genetic selection programs prioritizing fast growth and high breast yield have resulted in the emergence of growth-related myopathies in broiler chickens [1,2]. Spaghetti meat (SM), characterized by overall impaired muscle integrity with separation of the fibers [3], is the latest abnormality identified, leading to degenerative disorders in broiler muscle tissues. However, SM remains inadequately understood. In contrast, white striping (WS), marked by white striations of the *pectoralis major* muscle parallel to the fibers [4], and wooden breast (WB), characterized by fibrosis and *pectoralis major* hardness [5], have been subjected to extensive investigation [1,6,7]. Under commercial conditions, the occurrence of mild or moderate WS, severe WB, and SM can reach up to 96.0%, 11.8%, and 36.3%, respectively [8]. A recent meta-analysis of experimental data reported the highest occurrence of WS myopathy (64.7%), followed by SM (12%) and WB (11%) [9].

Severely affected meat faces consumer rejection and cannot always be marketed as fresh; it is redirected as raw material for processing or degraded to pet food [6,10]. Zanetti et al. [11] evaluated that in Brazil, approximately 0.8% of chicken breasts were condemned due to WB and WS, accounting for up to USD 70,632 in daily losses. In North America, the economic impact of these myopathies in broiler chickens was estimated to exceed USD 1 billion [12].

Defective *pectoralis major* meat shows changes in its chemical composition, reflected mainly in reduced protein levels, increased moisture and fat content [13,14,15], and altered fatty acid [16,17] and amino acid profiles [15,18] compared to normal breast meat. Although these changes are not severe enough to challenge the nutritional value of chicken meat—owing to their low absolute values—they can significantly impair the appearance and technological properties of the breast, such as color, texture, water holding, and binding capacity. Such impairments can dramatically limit the performance of the meat in further processed products, depending on the severity of the myopathy and the extent of structural changes [1,19,20]. Thus, although growth-related myopathies do not pose a public safety risk [17,21], they represent substantial economic losses [12] and a challenge to the sustainability of the broiler production chain [22].

The impact of SM, the most recently observed myopathy, on meat quality has been investigated to a lesser extent compared to WS and WB. Only limited reports are available on alterations in the fatty acid profile [14] and mineral composition [23] in SM, and currently, there are no data on the amino acid composition of SM. To our knowledge, only one study simultaneously examined the effects of these three main growth-related abnormalities on meat quality traits [14]. In addition, while Wang et al. [24] recently reported the combined effects of these myopathies on the textural and physical quality traits of chicken fillets, no comparative data are available regarding the differences in nutritional value and sensory attributes between WS, SM, WB, and normal meat.

Therefore, the present study aimed to provide a comprehensive evaluation of the effects of growth-related myopathies on the physical, chemical, nutritional, and sensory properties of chicken breast by assessing the technological, chemical, fatty acid, mineral, and amino acid compositions, as well as protein and lipid oxidation, in comparison to the sensory properties of normal breasts and *pectoralis major* muscles affected by WS, WB, and SM. The associated nutritional and health indices were also calculated.

## 2. Materials and Methods

### 2.1. Sampling of Chicken Breasts

The breasts used in the present study were obtained from our previous experiment, which addressed the dietary inclusion of different levels of *Chlorella vulgaris* biomass (0%, 3%, and 6%) in broiler chickens of both sexes kept in 36 pens under standard environmental conditions or submitted to chronic heat stress. The results related to the effects of experimental treatments on growth performance, carcass yields, meat quality, nutritional value of breast meat, and growth-related myopathies occurrence have been published by Bošković Cabrol et al. [25].

At the end of the experiment (broiler chickens at 42 days of age), all chickens were transported to a commercial poultry slaughterhouse, electrically stunned, and immediately slaughtered according to standard commercial practices. As detailed in the companion paper, 180 carcasses (5 per pen) were selected as representative in terms of average live weight and the variability of the corresponding pens and submitted to gross examination of pectoralis major for WS, WB [5,16], and SM occurrence [26]. Afterward, 108 carcasses (3 per pen) were stored in a refrigerated room at 4 °C. Then, 24 h post-mortem, the *pectoralis major* muscles were separated from the breasts, and the following analyses were performed:

– For the right *pectoralis major* muscle, the pH and color were observed (108 breasts; two per pen), and chemical analyses were performed (72 breasts; 2 per pen).
– For the left *pectoralis major* muscle, it was vacuum packaged, stored at –18 °C, and later used for measuring thawing and cooking losses (108 breasts; two per pen), texture (108 breasts; three per pen), and sensory characteristics (72 breasts; two per pen).

Out of the 108 carcasses analyzed, 55 had normal breasts (NORM), 26 had WS breasts, 9 exhibited WB, and 18 showed SM.

### 2.2. Quality and Technological Properties of Breast Meat

The pH of the breast meat was measured in triplicate on the ventral side using a pH meter (Basic 20, Crison Instruments S.A., Carpi, Italy) equipped with a specific electrode (cat. 5232, Crison Instruments S.A.).

As for color, instrumental color indices were assessed with a Minolta CM-508 C spectrophotometer (Minolta Corp., Ramsey, NJ, USA), utilizing illuminant 65D.

Thawing and cooking losses were evaluated according to the methods outlined by Petracci and Baéza [27].

Texture profile analysis (TPA) on cooked muscle was performed using a TA.HDI dynamometer (Stable Micro System Ltd., Goldaming, UK) with a 20 mm (diameter) cylindrical probe, moving with 5 mm compression (up to 40% of the original sample height) at a constant speed of 2 mm/s for two consecutive cycles, separated by a 5 s interval. Hardness (N), springiness (mm), cohesiveness, and chewiness (N × mm) were calculated using the Texture Exponent Connect software (Vers. 4.0.9.0; Stable Micro Systems Ltd., Surrey, UK). Hardness, cohesiveness, springiness and chewiness were determined according to Novaković and Tomašević [28].

The shear force of the cooked meat was measured using an LS5 dynamometer (Lloyd Instruments Ltd., Bognor Regis, UK) with an Allo–Kramer 10-blade probe (load cell: 500 kg; blade distance: 5 mm; blade thickness: 2 mm; cutting speed: 250 mm/min) [29,30].

### 2.3. Chemical, Amino Acid, Mineral and Fatty Acid Composition of the Breast Meat

The freeze-dried breast meat was ground and analyzed for proximate composition, which included dry matter (AOAC 934.01), ash (AOAC 967.05), crude protein (AOAC 2001.11), and ether extract (AOAC 991.36) content [31].

Amino acids, mineral and fatty acid composition of freeze-dried breast meat were determined as described in our companion paper [25].

### 2.4. Health, Nutritional, and Qualitative Indices

Health, nutritional, and qualitative indices, including the *n* − 6/*n* − 3 ratio, polyunsaturated FA/saturated FA ratio (PUFA/SFA), linoleic acid/α-linolenic acid ratio (LA/ALA), the sum of eicosapentaenoic acid and docosahexaenoic acid (EPA + DHA), unsaturation index (UI), nutrition value index (NVI), atherogenic index (AI), thrombogenic index (TI), hypocholesterolemic/hypercholesterolemic ratio (H/H), health-promoting index (HPI) and fish lipid quality/flesh lipid quality (FLQ), were calculated according to Dal Bosco et al. [32] as follows:

PUFA/SFA=(∑Polyunsaturated Fatty Acids)(∑Saturated Fatty Acids)

LA/ALA=(C18:2n−6)(C18:3n−3)

EPA+DHA=C20:5n−3+C22:6n−3

$$UI=\left(monoenoic\;FA\right)+(2\times dienoic\;FA)+(3\times trienoic\;FA)+(4\times tetraenoic\;FA)+(5\times pentaenoic\;FA)+(6\times hesaenoic\;FA)$$

NVI=(C18:0+C18:1n9)/(C16:0)

IA=[C12:0+(4×C14:0)+C16:0]/(∑UFA)

where UFA stands for unsaturated fatty acid

TI=(C14:0+C16:0+C18:0)/[(0.5×MUFA)+(0.5×∑PUFAn−6)+(3×∑ PUFAn−3)+(∑PUFAn−3/∑PUFAn−6)

where MUFA stands for monounsaturated fatty acid

H/H ratio=(C18:1+∑ PUFA)/(C12:0+C14:0+C16:0)

HPI=(∑ UFA)/[C12:0+(4×C14:0)+C16:0)]

FLQ=100×(C20:5n−3+C22:6n−3)/+(∑ SFA)

### 2.5. Lipid and Protein Oxidation of Breast Meat

Protein carbonylation in breast meat was determined according to a 2,4-dinitrophenylhydrazine (DNPH)-based method, as described by Levine et al. [33] modified by Soglia et al. [34] and described in detail in our companion paper [25].

Lipid oxidation was determined using the thiobarbituric acid reactive substances (TBARS) assay method, as described by Bao and Ertbjerg [35], with slight modifications as presented in the paper of Bošković Cabrol et al. [25].

### 2.6. Sensory Evaluation of Breast Meat

A quantitative descriptive sensory analysis (QDA) was performed on the *pectoralis major* muscle, involving twelve trained panelists (seven women and five men, aged 27–55 years) with three years of sensory evaluation experience. The panelists from the DAFNAE department were trained following ISO standards (ISO 3972, 5496, 8586) [36,37,38]. Over four weeks, 72 chicken breast samples (six per each of the 12 experimental groups) were evaluated in randomized order across seven sessions. Each session consisted of two sets of three samples. The panelists assessed attributes, including color, odor, taste, texture, and overall acceptability, on a 15-point continuous scale, with anchors at 0 (not intense) and 10 (very intense).

After 24 h of thawing at 4 °C, each meat sample was individually cooked in a vacuum-sealed bag in an 85 °C water bath for 25 min. The samples were then divided into four equal portions (3 cm × 2 cm × 1 cm), labeled with three-digit codes, and served in white plastic cups. To balance presentation order and minimize carryover effects, the samples were presented following a Williams Latin square design. Between samples, the panelists cleansed their palates with apple slices, unsalted crackers, and mineral water. Data collection was managed using Fizz v2.47b software (Biosystems, Couternon, France).

### 2.7. Statistical Analyses

Meat quality traits were first analyzed using an analysis of variance (ANOVA) with the PROC MIXED procedure in SAS (Vers. 9.4) [39], considering diet, environmental temperature, and sex as the main factors, along with their interactions, and treating the pen as a random effect. The corresponding results have been published and presented in the companion paper [25].

For the present study, when comparing the different types of defective meat with normal breast meat, the quality traits were submitted to ANOVA using the PROC GLM [39], with meat type (normal, WS, WB, and SM) as the main effect. The Bonferroni t-test was used to compare the least-square means. Differences among the means with *p* ≤ 0.05 were accepted as statistically significant.

## 3. Results and Discussion

The rate of myopathies observed in this study was moderate for WS (25.5%) and SM (18.3%) and low for WB (8.9%). These myopathy rates differ remarkably across studies, with WS ranging from 9.8% to 89.0% [40,41], SM from 6.2% to 89.2% [30,40], and WB from 16.7% to 37.3% [8,42]. The relatively low occurrence of WB in our samples can be considered a limitation of this study. Nevertheless, other research has also shown unbalanced sample sets due to the low occurrence or small number of WB (*n* = 5) cases [18,43] or normal breasts (only 4 normal breast samples out of 183 in 6-week-old broilers) [44]. It should be noted that the occurrence of WB and other myopathies in vivo is unpredictable and can only be confirmed post-mortem. A comparison across studies is difficult since myopathy rates can depend on several factors, e.g., body weight, breast yield, growth rate, and bird age [8,9,45,46]. It is worth highlighting that mild-to-moderate WS and WB were observed in the breast meat in the present study, whereas no severe WS or WB was found.

Regarding meat quality, the effects of myopathies on breast quality traits are presented in Table 1. Generally speaking, the presence of myopathies is known to impair meat quality by affecting its technological properties and limiting its suitability for further processing, with the extent of these effects depending on myopathy severity [1,19,20]. In the present study, the pH of the meat did not differ among the groups (*p* = 0.627), corroborating previous findings [3,47,48]. Regarding color, the presence of myopathies did not affect L* values (*p* = 0.670), as previously reported [47,49,50]. Nevertheless, the presence of WB reduced the a* index compared to WS (−74.5%), SM (−73.8%), and normal breasts (−73.1%) (*p* = 0.040), as well as the b* index compared to WS meat (−26.8%; *p* = 0.044). In contrast, some studies found that the redness of breast meat increased in the presence of WB [51,52,53,54], which can be attributed to the bulge and pale areas of WB-affected meat. On the other hand, other studies reported no effect on a* values [3,9,23,48]. Compared to normal meat, as shown in Table 1, WB meat had higher cooking losses (+13.7%; *p* = 0.002), whereas thawing losses did not differ among the groups (*p* = 0.736). The decrease in protein content (as reported in Table 2) and muscle fiber degeneration—which usually retain water—likely contributed to changes in thawing and cooking losses of defective meat [20]. Our findings are consistent with previous studies that identified increased cooking losses as an issue linked to WB [1,29,47]. Additionally, cooked WB samples exhibited higher shear force compared to SM samples (+19.9%; *p* = 0.041), with no differences between WB, SM, and normal meat. The TPA analysis (Table 1) did not reveal any changes in hardness, springiness, cohesiveness, or chewiness between the normal and defective meat, nor among the meat affected by the different myopathies (*p* > 0.05). Therefore, differences in texture according to the presence of myopathies depend on different factors. On the one hand, the increase in shear force measured in WB meat in comparison to SM is likely due to the greater cooking losses and collagen deposition in the former compared to the latter [14]. Therefore, SM is characterized by the rarefication of connective tissue, resulting in a softer texture, as previously reported in other studies [14,48]. FTIR spectroscopic and histological analyses found that the texture differences between WB meat and SM could be due to the differences in collagen fiber molecular structure and connective tissue organization. Specifically, collagen in the perimysium of WB-affected muscles is more abundant and consists of both thin and thick, randomly organized collagen fibers [55]. In contrast, SM is characterized by randomly organized, thinner, and looser immature collagen fiber bundles. In fact, Pascual et al. [48] found that (i) more force was needed to shear WB samples compared to SM samples using an Allo–Kramer blade (Texture Technologies Corp., Hamilton, MA, USA), as indicated by a larger area under the force deformation curve; and (ii) fewer peaks were recorded for WB compared to normal meat during the Meullenet–Owens razor shear (MORS) test, where peaks appear when a group of muscle fibers/connective tissue layers has been cut and, in the case of WB meat, the blade pushed and destroyed the muscle structure instead of cutting one layer at a time. On the other hand, in agreement with the present results, other studies [17,48,56] did not find a difference in shear force between WB and normal breast in the Allo–Kramer test, where cooking could reduce the differences between defective and normal meat [54]. Nevertheless, Chatterjee et al. [57] reported differences in some TPA traits (hardness and cohesiveness) between WB and normal breasts even after cooking, whereas in our study, the TPA analysis on cooked meat did not reveal differences according to meat type. Our findings confirm that shear force measurements on cooked meat may be more effective for discerning the texture differences between WB and SM, consistent with the findings of Pascual et al. [48].

The chemical composition of normal breasts and breasts with myopathies are shown in Table 2. The moisture, fat, and ash content did not differ among the meat types (*p* > 0.05), whereas the presence of WB reduced protein content compared to the other meat types (*p* < 0.001). Various studies have reported changes in the chemical composition of breasts with myopathies in terms of protein reduction [13,15,17,43,58], as found in the present study for WB meat, likely due to fibrosis causing the replacement of muscle fibers with connective and adipose tissue [13,14,18]. Other studies also observed reduced protein content in WS breasts [13], whereas, in the present study, protein content was similar in WS and normal meat, as previously reported by Soglia et al. [59] and Kuttappan et al. [16]. Prior studies reported a link between protein reduction and WS severity [16,60], where the low severity of WS recorded in the present study may account for the absence of differences in chemical composition in comparison with normal meat.

Indeed, changes in protein content in WB compared to normal meat are usually within a narrow range, i.e., 1.3–3.0 percentage units [15,17,40,58], with only one study [13] reporting larger changes (4.92 percentage units) in the protein content of WS/WB compared to normal meat. The changes observed in the present study are unlikely to have a meaningful impact on human nutrition for individuals consuming a balanced diet with a variety of protein sources, as the reduction in crude protein content is low (around 2 percentage points) and limited to WB. Additionally, Trithavisup et al. [61] evaluated the effect of WB on in vitro protein digestibility and cytotoxicity of cooked chicken breast meat and found no differences in free-NH_2_, the degree of hydrolysis, and the distribution of peptide molecular weight between normal and WB samples at late intestinal digestion, suggesting that WB has no effect on meat protein digestibility. However, the same authors suggested that further investigation is needed due to the higher WB content of peptides with oxidative modifications and reduced Caco-2 cell viability compared to normal samples, which could potentially have negative implications for human health.

In the literature, the reduction in protein content in defective meat has been accompanied by a decrease in isoleucine, leucine, and valine in WB [18] and in arginine, leucine, lysine, methionine, phenylalanine, threonine, and valine in WS/WB breasts compared to normal breasts [15]. In the present study, the presence of myopathies did not affect the amino acid composition (as displayed in Table 3), with the exception of glycine. Glycine content was higher in WB (*p* = 0.037), probably due to the higher content of the connective tissue rich in glycine [62] compared to breasts from other groups.

Regarding mineral composition, as presented in Table 4, the presence of myopathies had no impact on breast micro- and macro-element content (*p* > 0.05). However, some studies reported an alteration in mineral composition in breast meat affected by muscle abnormalities. Increased levels of calcium, sodium, and iron and reduced levels of potassium, magnesium, and phosphorus have been previously reported in WS breasts [13,50]. Moreover, WB breasts have shown lower levels of phosphorus and potassium and higher levels of aluminum, calcium, iron, sodium, and sulfur, while elevated levels of calcium and sodium were found in SM breasts compared to unaffected muscles [18]. In the present study, even if no significant differences in mineral content were found among the groups, Na content tended to increase from normal to WB fillets. Alteration in Na and Ca homeostasis likely has an impact on WB development [20,63], as, subsequent to the initial degeneration, the impairment of the sarcoplasmic reticulum results in an increased influx of calcium ions and the activation of calcium-dependent proteases, initiating myofiber necrosis [64].

Although none of the myopathies in the present study affected total fat content, the WB meat displayed some changes in fatty acid composition compared to the WS, SM, and normal breasts. As presented in Table 5, compared to the normal meat and SM, WB meat exhibited higher rates (*p* < 0.05) of C18:2*n*6 (+12.8% and +14.2%, respectively), C22:6*n*3 (+18.7% and +33.3%), *n*3 PUFA (+19.8% and +22.2%), *n*6 PUFA (+13.0% and +13.9%), and total PUFA (+13.6% and +14.6%), with the WS samples showing intermediate (*p* > 0.05) values. In addition, the rate of C18:3*n*6 was higher in WB meat than in normal meat (+24.6%; *p* < 0.01).

The suggested mechanisms for these results [17] are the presence of inflammatory cells with a relatively high proportion of arachidonic acid in their membrane phospholipids [65] or the tendency of fiber type switching from fast, glycolytic IIB fibers, in which lipids are generally more saturated, to those within slow and oxidative fibers [64,66]. There was a higher linolenic (ALA) and γ-linolenic (GLA) acid content in the WB breasts and a higher γ-linolenic acid content in the WS breasts compared to normal breasts in the present study, which could be due to inflammatory processes. Specifically, linoleic acid acts as a precursor to γ-linolenic acid and then arachidonic acid, the dominant substrate for proinflammatory eicosanoids synthesis [67]. Previous studies also reported increased linolenic and α-linolenic acid [16,17,43] content and total *n* − 3 and *n* − 6 fatty acids [17] in breasts affected by WB and WS myopathies, which is linked to sarcolemma damages and inflammatory processes. Consistent with our results, Soglia et al. [43] found no difference in MUFA content between WB, WS/WB, and normal breasts, whereas they reported a decrease in SFA content in muscles with abnormalities.

Further, as presented in Table 6, the differences in the nutritional indexes were not significant among the groups (*p* > 0.05), with the exception of the unsaturation index that differed between SM and WB meat (102 vs. 109; *p* = 0.05). The unsaturation index did not differentiate between *n* − 3 and *n* − 6 fatty acids, making it less specific for nutritional evaluation but important for assessing oxidative stability [68], where WB-affected breasts were likely more susceptible to lipid oxidation during storage.

**Figure 1:**
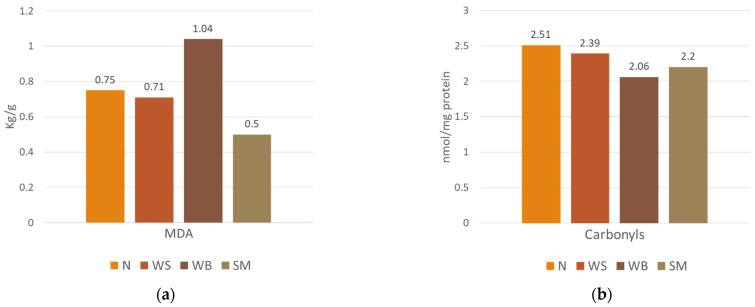
Lipid (**a**) and protein (**b**) oxidation changes in *pectoralis major* muscles affected (or not) by myopathies. N: normal meat; WS: white striping; WB: wooden breast; SM: spaghetti meat.

Regarding oxidative changes, MDA (*p* = 0.239) and carbonyl contents (*p* = 0.541) did not differ between normal meat and meat with WS, WB, and SM, nor among the defective meat samples, as presented in Figure 1. In this context, although no significant differences in lipid oxidation were found, the TBARS content was numerically higher in WB meat compared to normal meat and meat affected by WS and SM, likely due to somewhat increased PUFA portions. Other studies [43,69] reported WB presence promoting lipid oxidation in muscles due to the accumulation of reactive oxygen species and oxidative stress.

As reported in Table 7, the sensory attributes did not differ according to the presence of myopathies (*p*  > 0.05). The results of the present study are the first related to SM and show no difference in sensory attributes compared to normal or WS and WB meat. Tasoniero et al. [50] did not find differences concerning the perception of odor, strange taste, taste (sourness, bitterness, and aroma intensity), and juiciness between normal breasts and WS and WS/WB breasts; however, they did report an increase in the overall intensity of off-odors compared to normal breasts. On the other hand, several authors have reported significant detrimental effects of WB on texture, including increased hardness, as perceived by panelists [70,71]. In our study, these differences only approached statistical significance (*p* = 0.097). This does not imply a negative result per se, as Brazilian consumers preferred moderate and severe WB levels based on the taste, softness, juiciness, and chewiness of meat compared to normal breasts [53], suggesting that the effects of myopathies on final meat quality are also dependent on consumer preferences.

The inconsistency in results across studies may be attributed to differences in the severity of myopathies, the cooking processes applied, and sampling procedures, as certain structural changes and collagen deposition affect only the superficial muscle layers, whereas profound parts of the muscles remain intact [70,71,72]. In fact, Xing et al. [73] did not report a difference in sensory acceptance between raw normal and mild WB meat, whereas moderate and severe WB fillets affected the sensory evaluation of consumers owing to their impaired general appearance, texture, and drip losses, suggesting that the effect of WB is related to its grade (severity). Additionally, some authors [70,71] found that the effect of WB on meat sensory quality is not uniform throughout the breast and that the sensory attributes of springiness, hardness, and fibrousness were perceived differently between the ventral and dorsal sections of cooked WB breast meat.

## 4. Conclusions

Our findings indicate that while WB and SM significantly impact certain technological and chemical properties of meat—such as protein content, fatty acid composition, cooking loss, and texture—these alterations do not compromise the nutritional quality or sensory appeal of cooked meat for consumers. In other words, these myopathies affect processing suitability and may have economic implications, but mild-grade myopathies do not diminish the value of chicken meat for consumers. However, the potential impact of these muscle abnormalities on animal welfare remains a concern. Indeed, while consumer acceptance appears largely unaffected, examining preferences across different market segments and cultural contexts could provide valuable insights for both producers and policymakers. Finally, future studies should explore the biochemical pathways underlying each myopathy more deeply, focusing on preventive strategies at the farming level, such as optimized nutrition, environmental control, and genetic selection.

## Figures and Tables

**Table 1:** Quality traits of *pectoralis major* muscles in broiler chickens affected (or not) by myopathies.

	Meat Type				*p*-Value	RMSE
	NM	WS	WB	SM		
*Samples (n)*	55	26	9	18		
pH	5.88	5.91	5.89	5.91	0.627	0.11
Lightness (L*)	49.7	49.1	49.8	49.3	0.670	2.24
Redness (a*)	1.45 ^a^	1.53 ^a^	0.39 ^b^	1.49 ^a^	0.040	1.02
Yellowness (b*)	15.2 ^ab^	16.2 ^a^	11.8 ^b^	15.5 ^ab^	0.044	3.73
Thawing losses (%)	11.8	12.3	12.5	11.7	0.736	2.82
Cooking losses (%)	26.0 ^a^	27.5 ^ab^	30.1 ^b^	27.8 ^ab^	0.002	3.09
Shear force (N)	28.3 ^ab^	28.7 ^ab^	32.5 ^a^	26.0 ^b^	0.041	5.28
Hardness (N)	95.9	90.8	91.0	91.9	0.791	24.4
Springiness (mm)	1.60	1.67	1.74	1.58	0.581	0.36
Cohesiveness (N × mm)	0.60	0.59	0.60	0.59	0.847	0.06
Chewiness	91.1	89.7	95.7	87.7	0.943	32.2
Fat (%)	1.76	1.92	2.25	1.56	0.435	0.70

^a,b^ Different superscripts within a row indicate a significant difference (*p* ≤ 0.05). RSME: root mean square error, where SEM is equal to RMSE/√*n*, NM: normal meat; WS: white striping; WB: wooden breast; SM: spaghetti meat.

**Table 2:** Chemical composition of *pectoralis major* muscles in broiler chickens affected (or not) by myopathies.

	Meat Type				*p*-Value	RMSE
	NM	WS	WB	SM		
*Samples (n)*	39	21	3	9		
Moisture (%)	75.2	75.45	76.8	75.60	0.054	0.86
Ash (%)	1.14	1.13	1.10	1.13	0.496	0.05
Protein (%)	21.9 ^a^	21.5 ^a^	19.85 ^b^	21.71 ^a^	<0.001	0.72
Fat (%)	1.76	1.92	2.25	1.56	0.435	0.70

^a,b^ Different superscripts within a row indicate a significant difference (*p* ≤ 0.05). RSME: root mean square error where SEM is equal to RMSE/√*n*, NM: normal meat; WS: white striping; WB: wooden breast; SM: spaghetti meat.

**Table 3:** Amino acid profile (mg/100 g) of the *pectoralis major* muscles of broiler chickens affected or (or not) by myopathies.

	Meat Type				*p*-Value	RMSE
	NM	WS	WB	SM		
*Samples (n)*	39	21	3	9		
Histidine	908	921	1047	865	0.165	72.3
Hydroxyproline	380	411	435	390	0.322	48.6
Arginine	1130	1156	1223	1095	0.317	71.4
Serine	716	736	772	697	0.203	40.0
Glycine	742	790	837	717	0.037	54.3
Aspartic acid	1404	1436	1515	1344	0.333	101.0
Glutamic acid	2921	2982	3163	2787	0.272	200.9
Threonine	736	753	799	713	0.296	47.1
Alanine	876	904	839	839	0.169	58.0
Proline	566	594	627	544	0.057	37.6
Lysine	1477	1498	1603	1403	0.357	112.3
Methionine	477	483	509	468	0.552	27.3
Tyrosine	646	663	695	635	0.321	35.9
Valine	693	715	783	659	0.142	53.7
Cysteine	238	241	263	234	0.261	13.5
Isoleucine	673	690	756	647	0.227	50.8
Leucine	1245	1277	1359	1199	0.190	76.9
Phenylalanine	719	738	778	704	0.267	41.4
Tryptophan	203	203	198	208	0.702	9.57

RSME: root mean square error, where SEM is equal to RMSE/√*n*, NM: normal meat; WS: white striping; WB: wooden breast; SM: spaghetti meat.

**Table 4:** Mineral content (mg/100 g) in the *pectoralis major* muscles of broiler chickens affected (or not) by myopathies.

	Meat Type				*p*-Value	RMSE
	NM	WS	WB	SM		
*Samples* (*n*)	39	21	3	9		
Sodium	55.2	56.2	64.9	57.4	0.429	5.94
Potassium	369	365	353	363	0.403	12.01
Calcium	6.04	5.74	5.69	6.40	0.486	0.76
Magnesium	29.9	30.3	27.2	30.2	0.272	1.45
Phosphorus	233	232	216	235	0.216	7.88
Iron	0.41	0.40	0.38	0.42	0.519	0.04
Copper	0.040	0.034	0.037	0.042	0.613	0.01
Zinc	0.77	0.71	0.77	0.74	0.286	0.08
Manganese	0.011	0.011	0.011	0.011	0.958	0.002
Sulfur	190	190	184	192	0.461	4.71

RSME: root mean square error, where SEM is equal to RMSE/√*n*, NM: normal meat; WS: white striping; WB: wooden breast; SM: spaghetti meat.

**Table 5:** Fatty acid composition (g/100 g FAME) in the *pectoralis major* muscles of broiler chickens affected (or not) by myopathies.

	Meat Type				*p*-Value	RMSE
	NM	WS	WB	SM		
*Samples* (*n*)	39	21	3	9		
C14:0	0.55	0.55	0.62	0.55	0.109	0.04
C16:0	23.0	22.8	22.3	23.4	0.246	0.87
C17:0	0.15	0.15	0.16	0.14	0.851	0.02
C18:0	6.54	6.36	6.23	6.49	0.775	0.72
C20:0	0.14	0.13	0.14	0.12	0.226	0.02
Other SFA	0.73	0.73	0.72	0.73	0.999	0.08
C16:1*n*7	4.22	4.26	3.86	4.32	0.820	0.64
C16:1*n*9	0.44	0.46	0.48	0.43	0.095	0.04
C18:1*n*7	1.67	1.74	1.62	1.70	0.243	0.13
C18:1*n*9	33.3	33.1	30.8	33.1	0.054	1.22
Other MUFA						
C18:2*n*6 (LA)	24.9 ^a^	25.0 ^a^	28.1 ^b^	24.6 ^a^	0.043	1.56
C18:3*n*3 (ALA)	2.20	2.29	2.60	2.19	0.100	0.25
C18:3*n*6 (GLA)	0.27 ^a^	0.29 ^ab^	0.33 ^b^	0.27 ^ab^	0.009	0.03
C20:2*n*6	0.23	0.22	0.25	0.22	0.870	0.06
C20:4*n*6	0.60	0.68	0.70	0.64	0.154	0.14
C20:5*n*3	0.07	0.07	0.11	0.05	0.426	0.05
C22:5*n*3 (EPA)	0.16	0.19	0.18	0.16	0.200	0.04
C22:6*n*3 (DHA)	0.13	0.15	0.16	0.12	0.032	0.03
Other PUFA	0.26	0.31	0.32	0.30	0.098	0.08
Σ SFA	30.0	30.6	30.0	31.3	0.429	1.36
Σ MUFA	40.2	40.3	37.2	40.2	0.077	1.57
Σ PUFA	28.8 ^a^	29.2 ^ab^	32.7 ^b^	28.5 ^a^	0.035	1.88
*n*3	2.62	2.76	3.14	2.57	0.048	0.32
*n*6	26.2 ^a^	26.4 ^ab^	29.6 ^b^	26.0 ^a^	0.042	1.65

^a,b^ Different superscripts within a row indicate a significant difference (*p* ≤ 0.05). RSME: root mean square error, where SEM is equal to RMSE/√*n*. NM: normal meat; WS: white striping; WB: wooden breast; SM: spaghetti meat. SFA: saturated fatty acids. MUFA: monounsaturated fatty acids. PUFA: polyunsaturated fatty acids. LA: linoleic acid. ALA: α-linolenic acid. GLA: γ-linolenic acid. EPA: eicosapentaenoic acid. DHA: docosahexaenoic acid.

**Table 6:** Nutritional indexes of *pectoralis major* muscles in broiler chickens affected (or not) by myopathies.

	Meat Type				*p*-Value	RMSE
	NM	WS	WB	SM		
*Samples (n)*	39	21	3	9		
PUFA/SFA	0.93	0.96	1.09	0.91	0.074	0.09
*n*6*/n*3	10.07	9.67	9.42	10.22	0.217	0.89
LA/ALA	11.4	11.0	10.8	11.3	0.321	0.88
EPA + DHA (%)	0.20	0.22	0.28	0.18	0.162	0.07
Unsaturation index (UI)	103.2	104.5	109.1	102.7	0.050	3.35
Nutrition value index (NVI)	1.73	1.73	1.66	1.69	0.071	0.23
Atherogenic index (AI)	0.37	0.36	0.35	0.37	0.403	0.02
Thrombogenic index (TI)	0.73	0.71	0.68	0.74	0.249	0.05
Hypocholesterolemic to hypercholesterolemic ratio (H/H)	2.64	2.67	2.77	2.58	0.263	0.15
Health-promoting index (HPI)	2.74	2.78	2.82	2.69	0.386	0.15
Fish lipid quality/flesh lipid quality (FLQ)	0.65	0.74	0.93	0.57	0.128	0.246

RSME: root mean square error, where SEM is equal to RMSE/√*n*. PUFA: Polyunsaturated fatty acids. SFA: saturated fatty acids. LA: linoleic acid. ALA: α-linolenic acid. EPA: eicosapentaenoic acid. DHA: docosahexaenoic acid. NM: normal meat; WS: white striping; WB: wooden breast; SM: spaghetti meat.

**Table 7:** Sensory attributes in the *pectoralis major* muscles of broiler chickens affected (or not) by myopathies.

	Meat Type				*p*-Value	RMSE
	NM	WS	WB	SM		
*Samples* (*n*)	39	21	3	9		
Color	5.11	5.06	4.94	5.21	0.752	0.78
Flavor						
Brothy	4.28	4.07	3.81	3.95	0.171	1.05
Meaty/umami	5.57	5.46	5.25	5.47	0.743	1.05
Salty	3.49	3.27	3.62	3.22	0.250	0.99
Sweet	3.95	4.03	3.94	4.23	0.445	0.93
Abnormal taste	0.46	0.34	0.62	0.32	0.447	0.73
Abnormal odor	0.38	0.32	0.25	0.23	0.654	0.68
Overall acceptance	5.61	5.89	5.19	5.84	0.467	1.65
Texture						
Hardness	3.21	2.86	3.68	2.82	0.097	1.32
Juiciness	4.11	4.27	4.77	4.23	0.542	1.41
Chewiness	4.13	4.94	3.90	3.91	0.731	1.44
Cohesiveness	3.33	2.89	2.77	3.38	0.106	1.44
Fibrosity	4.71	4.77	3.99	4.70	0.471	1.31

RSME: root mean square error, where SEM is equal to RMSE/√*n*. NM: normal meat; WS: white striping; WB: wooden breast; SM: spaghetti meat.

## Data Availability

The data presented in this study are available upon request from the corresponding author.
